# Sulforaphane as a Potential Protective Phytochemical against Neurodegenerative Diseases

**DOI:** 10.1155/2013/415078

**Published:** 2013-07-25

**Authors:** Andrea Tarozzi, Cristina Angeloni, Marco Malaguti, Fabiana Morroni, Silvana Hrelia, Patrizia Hrelia

**Affiliations:** ^1^Department for Life Quality Studies, Alma Mater Studiorum, University of Bologna, Corso d'Augusto 237, 47900 Rimini, Italy; ^2^Department of Pharmacy and Biotechnology, Alma Mater Studiorum, University of Bologna, Via Irnerio 48, 40126 Bologna, Italy

## Abstract

A wide variety of acute and chronic neurodegenerative diseases, including ischemic/traumatic brain injury, Alzheimer's disease, and Parkinson's disease, share common characteristics such as oxidative stress, misfolded proteins, excitotoxicity, inflammation, and neuronal loss. As no drugs are available to prevent the progression of these neurological disorders, intervention strategies using phytochemicals have been proposed as an alternative form of treatment. Among phytochemicals, isothiocyanate sulforaphane, derived from the hydrolysis of the glucosinolate glucoraphanin mainly present in *Brassica* vegetables, has demonstrated neuroprotective effects in several in vitro and in vivo studies. In particular, evidence suggests that sulforaphane beneficial effects could be mainly ascribed to its peculiar ability to activate the Nrf2/ARE pathway. Therefore, sulforaphane appears to be a promising compound with neuroprotective properties that may play an important role in preventing neurodegeneration.

## 1. Introduction

Acute and chronic neurodegenerative diseases, including stroke, traumatic brain injury (TBI), Alzheimer's disease (AD), and Parkinson's disease (PD), are illnesses associated with high morbidity and mortality, and few or no effective options are available for their treatment [1, 2]. These diseases result in acute, as well as gradual and progressive neurodegeneration, leading to brain dysfunction and neuronal death. Although molecular mechanisms involved in the pathogenesis of acute and chronic neurodegenerative diseases remain elusive, oxidative stress, misfolding, aggregation, accumulation of proteins, perturbed Ca^2+^ homeostasis, excitotoxicity, inflammation, and apoptosis have been implicated as possible causes of neurodegeneration in the previously mentioned neurological disorders [3, 4]. In addition, recent studies demonstrated that acute brain injuries are also environmental risk factors associated with chronic neurodegenerative diseases [5–7].

In the last few years, there has been a growing interest in a number of pharmacological approaches aimed at preventing and counteracting the neuronal dysfunction and death associated with neurodegenerative diseases. However, while enormous efforts have been made to identify agents that could be used to alleviate debilitating neurodegenerative disorders, a source of potentially beneficial agents, namely, phytochemicals, would appear to have significant benefits in counteracting neurodegenerative diseases. Phytochemicals have long been recognized as exerting different biological effects, including antioxidant, antiallergic, antiinflammatory, antiviral, antiproliferative, and anticarcinogenic effects [8–10]. Considering that these age-related neurological disorders are multifactorial and that no drugs are available to stop their progression, intervention strategies using phytochemicals have been proposed as an alternative form of treatment for their prevention. Among phytochemicals, sulforaphane (isothiocyanato-4-(methylsulfinyl)-butane) (SF) has been demonstrated to have neuroprotective effects in several experimental paradigms. Reports in the literature have shown a pleiotropic role of this natural compound, thanks to its ability to address different targets and to modulate different pathways in neuronal/glial cells.

In this review, we will discuss the most recent experimental evidence on the role of SF in counteracting brain oxidative stress in both acute and chronic neurodegenerative diseases. SF bioavailability is also considered, since it is a fundamental aspect in the evaluation of the “in vivo” bioactivity of a nutritional compound.

## 2. Sulforaphane Bioavailability

Various Brassica vegetables and especially broccoli contain glucoraphanin. Following cutting or chewing, it is hydrolyzed into the corresponding isothiocyanate SF either by the plant thioglucosidase myrosinase or by bacterial thioglucosidases in the colon [11].

Because of its lipophilicity [12] and molecular size, SF is likely to passively diffuse into the enterocytes [13]. After absorption, SF is conjugated with glutathione (SF-GSH) by glutathione-S-transferase (GST) leading to maintenance of a concentration gradient and facilitating a fast passive absorption into the cell [14]. It is metabolized via the mercapturic acid pathway, producing predominantly cysteinylglycine (SF-CG), cysteine (SF-Cys), and N-acetyl-cysteine (SF-NAC) conjugates that are excreted in the urine [15].

Pharmacokinetic studies in both humans and animals showed that the plasma concentration of SF and its metabolites increased rapidly, reaching a maximum between 1 and 3 h after administration of either SF, glucosinolate, or broccoli [16–21]. In particular, Veeranki and colleagues [21] reported the ability of SF and its metabolites to reach different tissues in the gastrointestinal and genitourinary tracts and other organs such as liver, pancreas, lung, and heart, in vastly different concentrations and that bioactivity, in terms of induction of cytoprotective phase II enzymes, may differ significantly among organs. Both plasma and tissue levels of these SF metabolites are rapidly eliminated through urinary excretion within 12–24 h reflecting the rapid elimination of SF. The in vivo bioactivity of each SF metabolite is still unclear, although many in vitro studies have shown the ability of SF-Cys, and SF-NAC metabolites to exert some bioactivity [22–24]. These data suggest the hypothesis that repeated consumption of SF or cruciferous vegetables is required to maintain the SF metabolite concentration in tissues. 

Interestingly, more recent SF bioavailability studies in human subjects consuming broccoli showed its bioconversion into isothiocyanate erucin (isothiocyanato-4-(methylthio)-butane) (ER), a sulfide analog [25, 26]. Whether this conversion from SF to ER is important for the health promoting effects of glucosinolate still remains to be determined although some reports provide a glimpse into the possibility of differing activities between these two isothiocyanates [27–29]. 

In order to exert protective effects towards neurodegenerative disorders or improve brain function, SF must traverse the blood-brain barrier (BBB) and accumulate in the central nervous system (CNS). As reported in the following sections of this review, various studies in animal models of neurodegeneration suggest the ability of SF to reach CNS and to display protective effects at this level. In this context, Jazwa et al. [30] demonstrated in mice that after SF gavage, SF is able to cross the BBB and to accumulate in cerebral tissues such as the ventral midbrain and striatum, with a maximum increase and disappearance after 15 min and 2 h, respectively. Interestingly, Clarke et al. [19] also detected SF-GSH, SF-Cys and SF-NAC metabolites, but not SF alone, in the CNS in a similar experimental in vivo model after 2 h and 6 h. However, the authors suggest that low levels of the various SF metabolites recorded in the CNS indicate their poor ability to cross the BBB. These results show the ability of SF to quickly reach the CNS and the potential contribution of SF metabolites to prolong the presence of SF at this level because they are unstable under physiological conditions and readily dissociate back to SF [21, 30]. 

## 3. Protective Effects of Sulforaphane against Oxidative Stress

Oxidative stress results from an imbalance of pro-oxidant/antioxidant homeostasis that leads to an abnormal production of reactive oxygen species (ROS) and reactive nitrogen species (RNS). The main ROS/RNS involved in neurodegeneration are superoxide anion radical (O_2_^•−^), hydrogen peroxide (H_2_O_2_), the highly reactive hydroxyl radical (^•^OH), and nitric oxide (NO) that can react with superoxide anion to produce peroxynitrite [31]. At high levels, ROS can react with different cell molecules, causing damage to DNA, lipids, and proteins and modulate intracellular signaling pathways, leading to cellular degeneration and apoptosis. ROS can also initiate proinflammatory pathways, further exacerbating the deleterious oxidized environment. The brain is particularly vulnerable to oxidative stress because of its high oxygen consumption, high content of oxidizable polyunsatured fatty acids, and low antioxidant defense capacities especially in aging brains [32–34]. Oxidative stress is involved in many neurodegenerative diseases and is a proposed mechanism for age-related degenerative processes as a whole [35, 36]. Numerous studies have provided compelling evidence that oxidative stress is an important causative factor in PD [2, 37–40], AD [41–43], amyotrophic lateral sclerosis (ALS) [44, 45], and multiple sclerosis (MS) [46, 47]. 

Cells possess a complex network of nonenzymatic and enzymatic components to counteract oxidative stress. GSH is the major nonenzymatic regulator of intracellular redox homeostasis. On the other hand, enzymatic antioxidants include glutathione S-transferase (GST), glutathione reductase (GR), glutathione peroxidase (GPx), NAD(P)H-quinone oxidoreductase 1 (NQO1), thioredoxin reductase (TR), heme oxygenase 1 (HO1), peroxiredoxins, and many others. These enzymes are now recognized as primary defense mechanisms against many degenerative and chronic disease conditions [48]. These antioxidants and cytoprotective enzymes are regulated by a common mechanism that involves two proteins: nuclear factor erythroid 2-related factor 2 (Nrf2) and Kelch-like-ECH-associated protein 1 (Keap1) [49, 50]. Under basal conditions, Nrf2 is sequestered in the cytoplasm by its repressor protein Keap1 [51]. Keap1 contains several reactive cysteine residues that serve as sensors of the intracellular redox state. Nrf2 is released from Keap1 upon oxidative or covalent modification of thiols in some of these cysteine residues. Nrf2 translocates to the nucleus where it heterodimerizes with small Maf proteins before binding to the antioxidant responsive element (ARE) [35, 52] within the promoter regions of many cytoprotective genes [36]. In addition, Nrf2 has a key role against inflammation thanks to its ability to antagonize the transcription factor nuclear factor-*κ*B (NF-*κ*B) which regulates the expression of inflammatory genes [37].

ARE induction by chemical activators has been shown to protect neuronal cell lines against various oxidative damages induced by dopamine, hydrogen peroxide (H_2_O_2_), and glutamate [38–40]. SF has been demonstrated to increase many ARE-dependent antioxidant enzymes in different cell systems [41–43], such as GR, GPx, glutaredoxin (GLRX), thioredoxin (TX), TR, HO1, and NQO1. It has been shown that SF directly interacts with Keap1 by covalent binding to its thiol groups [44]. 

Negi et al. [45] demonstrated that SF increased the expression of Nrf2 and of downstream targets HO-1 and NQO-1 in Neuro2a cells and the sciatic nerve of diabetic animals. SF was also effective in counteracting oxidative stress induced by antipsychotic drugs in human neuroblastoma SK-N-SH cells, increasing GSH levels and inducing NQO1 activity [46].

Sulforaphane prevented oxidative stress-induced cytotoxicity in rat striatal cultures by raising the intracellular GSH content via an increase in *γ*-GCS expression induced by the activation of the Nrf2-antioxidant responsive element pathway [47]. 

It has also been observed that oxidative stress can inactivate peroxiredoxins, an important family of cysteine-based antioxidant enzymes that exert neuroprotective effects in several models of neurodegeneration [48, 53–55]. Interestingly, in both neurons and glia, SF treatment upregulates sulfiredoxin, an enzyme responsible for reducing hyperoxidized peroxiredoxins [56]. SF pretreatment also leads to attenuation of the tetrahydrobiopterin (BH4) induced ROS production thanks to the increase in mRNA levels and enzymatic activity of NQO1 in DAergic cell lines CATH.a and SK-N-BE(2)C [57].

Kraft et al. [58] demonstrated the importance of ARE activation in astrocytes of a mixed primary culture system. They observed that SF induced an ARE-mediated genetic response that is highly selective for astrocytes over neurons and conveys neuroprotection from oxidative insults initiated by H_2_O_2_ or nonexcitotoxic glutamate toxicity. Innamorato et al. [59] observed a direct association between the protective effect of SF against oxidative stress induced by lipopolysaccharide with HO-1 induction in BV2 microglial cells. 

Oxidative stress induces Ca^2+^-dependent opening of the mitochondrial inner membrane permeability transition pore (PTP), causing bioenergetic failure and subsequent death in different cell models, including those related to acute brain injury [60–62]. Intraperitoneal injection of rats with a nontoxic level of SF resulted in resistance of isolated nonsynaptic brain mitochondria to peroxide-induced PTP opening [63], and this could contribute to the neuroprotection observed with SF.

BBB damage following oxidative stress has been extensively investigated [64]. Postinjury induction of Nrf2-driven genes by SF treatment attenuated the loss of endothelial cells and tight junction proteins and reduced BBB permeability and cerebral edema [65]. Another study demonstrated that SF administration reduced BBB permeability in a rat subarachnoid hemorrhage model likely through the antioxidative effects of the activated Nrf2-ARE pathway [66].

Less attention has been focused on oxidative damage at the blood-cerebrospinal fluid (CSF) barrier (BCSFB) located at the choroid plexus (CP) epithelium. Even modest changes in the CPs may have a marked impact on the brain. For example, changes in CP function have been implicated in Alzheimer's disease [67]. A study by Xiang et al. [68] demonstrated that SF can protect the BCSFB in vitro from damage caused by H_2_O_2_ and reduced H_2_O_2_-induced cell death in primary CP epithelial cells and a CP cell line Z310. 

**Figure 1:**
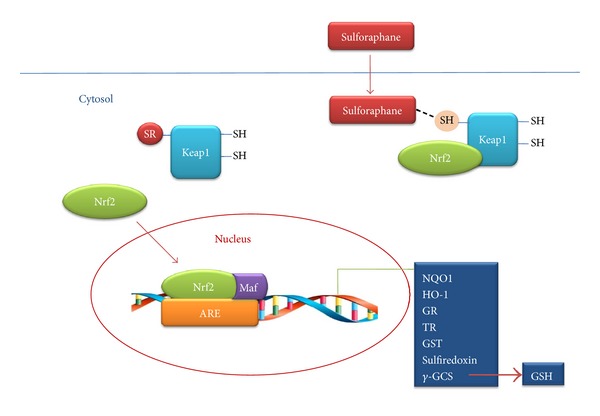
Proposed mechanism of neuroprotective effects provided by SF through Keap1/Nrf2 transcriptional activation of the antioxidant system. Adapted from [124].

Summarizing, the observed protective effects of SF against brain oxidative stress are mainly associated with Nrf2 activation and the resulting upregulation of antioxidant cytoprotective proteins and elevation of GSH (Figure 1).

## 4. Protective Effects of Sulforaphane against Acute Neurodegeneration

### 4.1. Ischemic Brain Injury

The pathophysiology of ischemic brain injury involves various biochemical mechanisms, such as glutamate-mediated excitotoxicity, the generation of ROS, apoptosis, and inflammation [69]. In adults, brain ischemic insults typically result from stroke or cardiac arrest, while in infants, cerebral ischemia is mediated by complications during labor and delivery, resulting in neonatal hypoxic-ischemic encephalopathy. In both groups, restoring blood flow to the ischemic brain is essential to salvage neurons. However, reperfusion itself causes additional and substantial brain damage referred to as “reperfusion injury.” 

In a neonatal hypoxia/ischemia brain injury model, Ping et al. [70] observed that SF significantly increased Nrf2 and HO-1 expression which was accompanied by reduced infarct volume. In particular, SF treatment reduced the number of apoptotic neurons, activated macroglia, and some oxidative parameters such as the amount of 8-hydroxy-2-deoxyguanosine and MDA level. In a similar model of ischemia/reperfusion induced by either oxygen and glucose deprivation or hemin in immature mouse hippocampal neurons, SF treatment activated the ARE/Nrf2 pathway of antioxidant defenses and protected immature neurons from delayed cell death [71]. Zhao et al. [69] demonstrated that delayed administration of a single dose of SF significantly decreased cerebral infarct volume in rats following focal ischemia. Moreover, in rat cortical astrocytes, SF treatment before or after oxygen and glucose deprivation significantly reduced cell death, stimulating the Nrf2 pathway of antioxidant gene expression [72]. In contrast to these data, Porritt et al. [73] showed that SF treatment initiated after photothrombosis-induced permanent cerebral ischemia in mice did not interfere with key cellular mechanisms involved in tissue damage. The authors suggest that the small volume of infarcted cortical tissue resulting from the photothrombosis injury might result in the generation of relatively smaller amounts of ROS and may explain why they did not observe any neuroprotection after SF administration. In addition, Srivastava et al. [74] recorded that the pretreatment of rats with SF decreased the nuclear accumulation of Nrf2 following cerebral ischemia/reperfusion injury. On this topic, the authors speculate that rapid accumulation of SF in the brain and subsequent upregulation of Nrf2 and antioxidant enzymes may reduce the need for the later adaptive increase in Nrf2 expression following stroke.

These lines of evidence indicate that SF may counteract ischemia/reperfusion due to its ability to modulate Nrf2 and intracellular redox signaling.

### 4.2. Traumatic Brain Injury

Traumatic brain injury (TBI) is defined as damage to the brain caused by external mechanical force [75]. Survivors of TBI are left with long-term disabilities, and even a mild TBI can leave people with cognitive impairments, difficulty in concentrating, headaches, and fatigue [76]. TBI is a complex disease process [77] that results in early phase of mechanical damage of brain tissue and a secondary phase of cellular and molecular events that cause oxidative damage and brain cell death [78, 79]. Despite advances in prevention measures, surgical, and diagnostic techniques, no pharmacological treatment has so far been found to confer neuroprotection by targeting secondary injury mechanisms [76]. 

Recent studies in a rat model of TBI showed that postinjury administration of SF reduces the BBB impairment and cerebral edema after TBI [65, 80]. In particular, Zhao et al. [80] showed that SF attenuated aquaporin-4 (AQP4) channel loss in the injury core and further increased AQP4 protein levels in the penumbra region at 24 h and 3 days following TBI. In contrast to the early increase of AQP4 levels, the decrease in cerebral edema was observed only at 3 days, confirming the important role of AQP4 channels to clear the water in excess and to maintain the brain water homeostasis [81]. However, the authors suggest that the observed SF neuroprotective effect may be due to a combination of mechanisms that include decreased BBB permeability, enhanced cell survival, and/or increased AQP4 channel levels. In particular, the restoration of AQP4 channel activity prevented the impaired clearance of extracellular potassium with neuronal depolarization and glutamate release. It should be noted that the glutamate release is involved in an important sequel of CNS injury [80]. In the same rat model of TBI, Zhao et al. [65] demonstrated that postinjury administration of SF preserved BBB function through the reduction of endothelial cell markers and tight junction protein loss. These protective effects were mediated by the activity of Nrf2. In particular, SF increased the expression of Nrf2-driven cytoprotective genes such as GST*α*3, GPx, and HO-1 in the parietal cortex and brain microvessels. More recent papers confirmed these findings in both rat and mice models of TBI [82]. Interestingly, Dash et al. [83] showed that in addition to vascular protection of SF, postinjury SF treatment preserved neurological function in injured animals. This improvement was demonstrated by enhanced learning and memory and by improved performance in a working memory task. The authors propose that the ability of SF to improve the hippocampal- and prefrontal cortex-dependent cognitive function could be ascribed to its ability to protect the neurons and other cell types of the neurovascular unit from the oxidative damage elicited by TBI. Taken together, these findings suggest that SF may protect against the various pathophysiological consequences of TBI and other neurological traumatic injuries. On this topic, a recent study demonstrated that SF provides neuroprotective effects in the spinal cord after contusive injury [84].

## 5. Protective Effects of Sulforaphane against Chronic Neurodegeneration

### 5.1. Alzheimer's Disease

Alzheimer's disease (AD) is the most common neurodegenerative disease that accounts for most cases of dementia experienced by older people and is characterized by a progressive decline in memory and impairment of at least one other cognitive function [85]. 

This neurodegenerative disease is characterized by the accumulation of amyloid beta (A*β*) peptides that result in oxidative damage, inflammation and increased intracellular calcium levels [86, 87]. Two major hallmarks of AD are the extracellular aggregation of A*β* peptides and the intracellular precipitation/aggregation of hyperphosphorylated Tau (forming neurofibrillary tangles) protein [87]. In particular, A*β* 1–40 and A*β* 1–42 peptides, produced by the cleavage of the precursor protein, can exist in multiple aggregation forms, including soluble oligomers or protofibrils, and insoluble fibrils, which are responsible for various pathological effects [88, 89].

Several studies showed that increased oxidative stress, the impaired protein-folding function of the endoplasmatic reticulum, and deficient proteasome- and autophagic-mediated clearance of damaged proteins accelerated the accumulation of A*β* peptides and Tau protein in AD [90, 91].

In this context, Kwak et al. [92] demonstrated that the neuroprotective effects of SF against oxidative stress, in terms of protein carbonyl formation and cytotoxicity elicited by hydrogen peroxide, could be ascribed to its ability to induce proteasome expression in murine neuroblastoma Neuro2A cells. In similar cellular models, Park et al. [93] confirmed the ability of SF to enhance the proteasome activities and to protect the neuronal cells from A*β*1–42-mediated cytoxicity. More recent studies reported that SF induced the expression of heat shock protein 27, demonstrating that SF-stimulated proteasome activity may contribute to cytoprotection [94]. These data suggest that induction of proteasome by SF may facilitate the clearance of the A*β*1–42 peptides and lead to the improvement of protein misfolding in AD. Kim et al. [95] investigated the potential neuroprotective effects of SF in an A*β*1–40 peptide-induced AD acute mouse model. In particular, they recorded the ability of SF to ameliorate the cognitive function impairment although it did not directly interact with A*β*. These findings reinforce the indirect neuroprotective effects of SF against A*β* toxicity.

### 5.2. Parkinson's Disease

Parkinson's disease (PD) is an age-related neurodegenerative disease with progressive loss of dopaminergic (DA) neurons in the substantia nigra pars compacta and with accumulation of neuronal inclusions known as Lewy bodies [96]. The exact etiology of PD remains to be fully elucidated, but the most reliable theories propose either an environmental [97, 98] or a genetic [99] origin, or a combination of both. Genetic studies have demonstrated that *α*-synuclein protein, a principal component of Lewy body inclusions [100], is a key participant in the pathogenesis of this disorder [101–103]. The exact biological function of *α*-synuclein and the mechanism by which mutations in this gene lead to neuron loss are still not clear, although it has been observed that an excess of *α*-synuclein protein can cause DA neuron loss [104].

Overwhelming evidence indicates that oxidative damage induced by ROS participates in the progression of DA neurons. In particular, the metabolism of dopamine (DA) might be responsible for the high basal levels of oxidative stress in the SN. Autooxidation of dopamine leads to the formation of neurotoxic species such as electrophilic DA quinone and ROS including superoxide anion (O_2_^•^) and H_2_O_2_ [105]. DA quinone is also thought to cause mitochondrial dysfunction [106] and to mediate *α*-synuclein-associated neurotoxicity in PD by covalently modifying *α*-synuclein monomer [107] and by stabilizing the toxic protofibrillar *α*-synuclein [108].

Using a *Drosophila* model of *α*-synucleinopathy, Trinh et al. [109] observed that the neuronal death accompanying *α*-synuclein expression is enhanced by loss-of-function mutations in genes involved in the phase II detoxification pathway, specifically, glutathione metabolism. This neuronal loss can be overcome by pharmacological inducers, including SF, that increase glutathione synthesis or glutathione conjugation activity. They also observed similar neuroprotective effects of SF in *Drosophila* parkin mutants, another loss-of-function model of PD.

Several in vitro studies showed that SF was able to significantly reduce DA quinone levels in dopaminergic cell lines, such as CATH.a and SK-N-BE(2)C, as well as in mesencephalic dopaminergic neurons, evoked by 6-hydroxydopamine (6-OHDA) and BH4 [110]. In particular, Han et al. [57] demonstrated that SF can protect dopaminergic cells from the cytotoxicity of 6-OHDA and BH4 by removal of intracellular DA quinone, because NQO1 enzyme activity and mRNA level are increased by SF treatment and quinone-modified proteins are decreased. 

**Figure 2:**
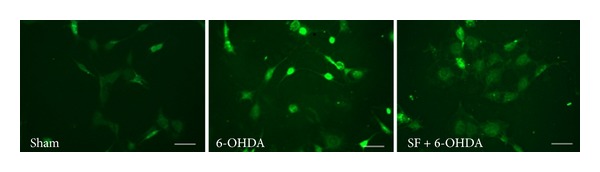
SF prevents 6-OHDA-induced ROS formation in SH-SY5Y cells. Representative images of SH-SY5Y cells incubated with SF for 24 h and then treated with 6-OHDA for 3 h. At the end of incubation, ROS formation was determined by fluorescence probe, 2′,7′-dichlorofluorescein-diacetate (DCFH-DA). Scale bar: 100 *μ*m.

In addition, DA quinone may yield neurotoxic species following its reaction with cellular thiols to form the 5-S-cysteinyl-dopamine (CysDA) [111–113]. CysDA adducts have been reported in human brain tissue and are elevated in the brains of patients suffering from PD [114]. We have demonstrated that SF is able to protect primary cortical neurons against CysDA-induced injury. In particular, we found that the protection exerted by SF against this neurotoxin is linked to the activation of ERK1/2, to the associated release of Nrf2 from Keap1, and to a subsequent increase in the expression and activity of specific detoxifying phase II enzymes [115]. Moreover, we demonstrated that SF prevented the dopaminergic-like neuroblastoma SH-SY5Y cell death, in terms of apoptosis and necrosis, induced by oxidant compounds, such as H_2_O_2_ and 6-OHDA, by its abilities to increase endogenous GSH, enzymes involved in GSH metabolism including GST and GR, and to normalize the intracellular redox status (Figure 2) [116]. Interestingly, we recorded similar in vitro neuroprotective effects also with the erucin generated by bioconversion of the SF suggesting a neuroprotective role of SF metabolites in PD [117].

Deng et al. [118] observed that SF inhibited 6-OHDA-induced cytotoxicity in SH-SY5Y cells through increasing Nrf2 nuclear translocation and HO-1 expression in a PI3 K/Akt-dependent manner. Further, other authors confirmed that Nrf2 activation by SF may play an important role in DA neuron protection against 6-OHDA-induced toxicity in rat organotypical nigrostriatal cocultures [119]. As regards in vivo neurodegeneration models, Jazwa et al. [30] demonstrated that SF induced an Nrf2-dependent phase II response in the basal ganglia and protected against nigral dopaminergic cell death, astrogliosis, and microgliosis in the 1-methyl-4-phenyl-1,2,3,6-tetrahydropyridine mouse model of PD. Further, we reported the ability of SF to exert neuroprotective effects on DA neurons in 6-OHDA-lesioned mice. In particular, these effects may be attributed to SF ability to enhance GSH levels and its dependent enzymes, including GST and GR, and to modulate neuronal survival pathways, such as ERK1/2 [120]. 

## 6. Conclusions

Several in vitro and in vivo studies have demonstrated the ability of SF to prevent various neurodegenerative processes that underlie stroke, traumatic brain injury, AD, and PD. The ability of SF to exert neuroprotective effects in different acute and chronic neurodegenerative diseases could be ascribed to its peculiar ability to activate the Nrf2/ARE pathway. Nrf2 is a recent therapeutic target in neurodegenerative diseases because it regulates several genes that have been implicated in protection against neurodegenerative conditions [121, 122]. In this context, SF presents many advantages, such as good pharmacokinetics and safety after oral administration as well as the potential ability to penetrate the BBB and deliver its neuroprotective effects in the central nervous system [123]. Based on these considerations, SF appears to be a promising compound with neuroprotective properties that may play an important role in preventing neurodegenerative diseases.
